# Echocardiography in Pediatric Pulmonary Hypertension

**DOI:** 10.3389/fped.2014.00124

**Published:** 2014-11-12

**Authors:** Pei-Ni Jone, D. Dunbar Ivy

**Affiliations:** ^1^Pediatric Cardiology, Children’s Hospital Colorado, University of Colorado School of Medicine, Aurora, CO, USA

**Keywords:** pediatric pulmonary hypertension, echocardiography, right heart, right ventricular function

## Abstract

Pulmonary hypertension (PH) can be a rapidly progressive and fatal disease. Although right heart catheterization remains the gold standard in evaluation of PH, echocardiography remains an important tool in screening, diagnosing, evaluating, and following these patients. In this article, we will review the important echocardiographic parameters of the right heart in evaluating its anatomy, hemodynamic assessment, systolic, and diastolic function in children with PH.

## Introduction

Pulmonary hypertension (PH) is a progressive disease that carries high morbidity and mortality. Although cardiac catheterization is used to define PH, echocardiography is the most important non-invasive tool that is used to detect PH (1). It provides the anatomy of the right heart, non-invasive hemodynamic assessment, systolic and diastolic evaluation of the right heart, and serial follow-up for this patient population. A diagnostic classification has been developed and modified at the World Symposiums on Pulmonary Hypertension (WSPH). Initially developed at the second WSPH in Evian (France) in 1998, this clinical classification system identifies five categories of disorders that cause PH, with each group sharing similar hemodynamic, pathologic, and management features; Pulmonary arterial hypertension (PAH) (Group 1); PH due to left heart disease (Group 2); PH due to chronic lung disease and/or hypoxia (Group 3); chronic thromboembolic PH (Group 4); and PH due to multifactorial mechanisms (Group 5) Table 1 (1). Echocardiography is valuable in each of these disorders as will be described. We will further discuss the conventional and advanced echocardiographic evaluation of pediatric PH.

**Table 1:** **Updated classification of pulmonary hypertension**.

1. Pulmonary arterial hypertension
1.1 Idiopathic PAH
1.2 Heritable PAH
1.2.1 BMPR2
1.2.2 ALK-1, ENG, SMAD9, CAV1, KCNK3
1.2.3 Unknown
1.3 Drug and toxin induced
1.4 Associated with:
1.4.1 Connective tissue disease
1.4.2 HIV infection
1.4.3 Portal hypertension
1.4.4 Congenital heart diseases
1.4.5 Schistosomiasis
1’ Pulmonary veno-occlusive disease and/or pulmonary capillary hemangiomatosis
1”. Persistent pulmonary hypertension of the newborn (PPHN)
2. Pulmonary hypertension due to left heart disease
2.1 Left ventricular systolic dysfunction
2.2 Left ventricular diastolic dysfunction
2.3 Valvular disease
2.4 Congenital/acquired left heart inflow/outflow tract obstruction and congenital cardiomyopathies
3. Pulmonary hypertension due to lung diseases and/or hypoxia
3.1 Chronic obstructive pulmonary disease
3.2 Interstitial lung disease
3.3 Other pulmonary diseases with mixed restrictive and obstructive pattern
3.4 Sleep-disordered breathing
3.5 Alveolar hypoventilation disorders
3.6 Chronic exposure to high altitude
3.7 Developmental lung diseases
4. Chronic thromboembolic pulmonary hypertension (CTEPH)
5. Pulmonary hypertension with unclear multifactorial mechanisms
5.1 Hematologic disorders: chronic hemolytic anemia, myeloproliferative disorders, splenectomy
5.2 Systemic disorders: sarcoidosis, pulmonary histiocytosis, lymphangioleiomyomatosis
5.3 Metabolic disorders: glycogen storage disease, Gaucher disease, thyroid disorders
5.4 Others: tumoral obstruction, fibrosing mediastinitis, chronic renal failure, segmental PH

*BMPR: bone morphogenic protein receptor type II; CAV1: caveolin-1; ENG: endoglin; HIV: human immunodeficiency virus; PAH: pulmonary arterial hypertension*.

## Conventional Two-Dimensional Echocardiography

Two-dimensional (2D) echocardiography provides qualitative and quantitative evaluation of the severity of PH.

### Right atrium

**Figure 1:**
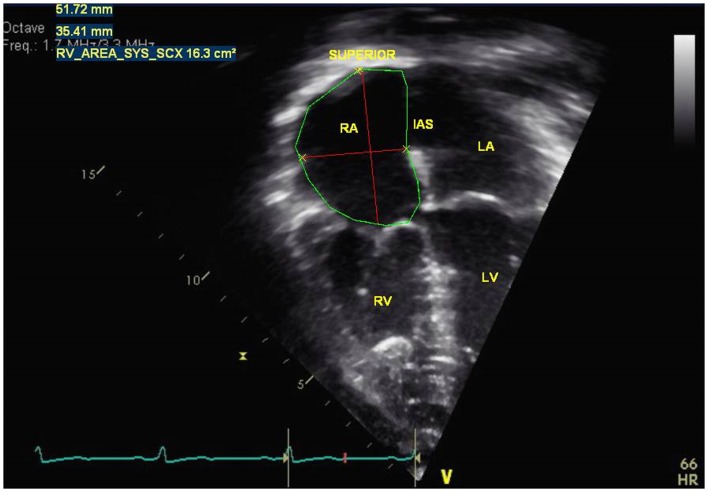
**Measurement of right atria dimensions (major and minor axis) and right atrial area in end-systole in a pulmonary hypertension patient**. RA = right atria, RV = right ventricle, IAS = interatrial septum, LA = left atria, LV = left ventricle.

The right atrium (RA) dilates over time in patients with PH and it represents decreased right ventricular (RV) compliance and RV diastolic dysfunction. It is a reservoir for systemic venous return when the tricuspid valve is closed, a passive conduit during early diastole, and active conduit in late diastole (2). Imaging of the RA is easily obtained in the apical four chamber view where RA dimensions of minor and major axis are measured and planimetry of the RA area in end-systole is performed to evaluate for RA dilation (Figure 1) (3). Indexed RA area to body surface area in adult patients with idiopathic PH has been a predictor of mortality and has been shown to be a prognostic marker for follow up of PH patients in adults and children (4–6).

### Inferior vena cava dilation

The inferior vena cava (IVC) can be dilated in patients with PH because of rising RA pressure. It is measured in the subcostal longitudinal view with IVC entering the RA. RA pressure can be estimated by IVC diameter and the presence of inspiratory collapse (7–9). IVC diameter ≤2.1 cm that collapses > 50% with a sniff suggests a normal RA pressure of 3 mmHg (range, 0–5 mmHg). IVC diameter ≥ 2.1 cm that collapses <50% with a sniff suggests a high RA pressure of 15 mmHg (range 10–20 mmHg) (3, 8, 10, 11). In children, the IVC varies with age of the patient. Elevated RA pressure in children can be assessed on the percentage of collapse of the IVC during inspiration rather than an absolute number.

### Right ventricle

**Figure 2:**
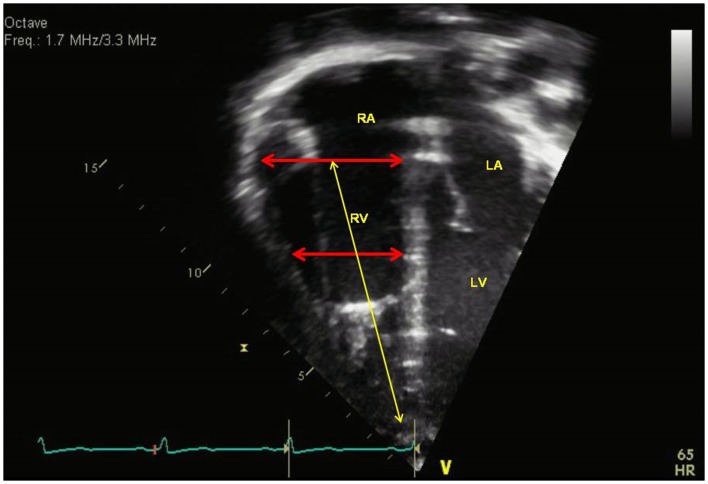
**Measurement of right ventricular dimensions in end-diastole with basal dimension at the level of the tricuspid valve, the mid-cavity dimension (red arrows), and longitudinal dimension (yellow arrow)**. RA = right atria, RV = right ventricle, LA = left atria, LV = left ventricle.

With chronic pressure overload, there is progressive hypertrophy and dilation of the RV. The complex morphology of the RV makes 2D imaging of the RV difficult and frequently requires multiple views in the parasternal, apical, and subcostal views to completely evaluate the RV dimensions. However, current recommendation from the guidelines in assessing the right heart includes a standardized imaging of the RV linear dimensions to evaluate for RV dilation (3). The RV size can be measured from the apical four chamber view at end-diastole in the “RV-focused view” (3, 12). The basal diameter is measured at the level of the tricuspid valve and the mid-cavity diameter is measured at the middle third of the RV at the level of the left ventricular (LV) papillary muscle. The longitudinal dimension is from the plane of the tricuspid valve annulus to the RV apex (Figure 2). Indexed RV end-diastolic diameter measured just above the tricuspid valve annulus reported by Burgess et al. has been associated with poor prognosis in patients with chronic pulmonary disease in adults (13). RV dilation is an early sign of RV maladaptation to increased pressure overload and an early sign of RV dysfunction (14). This has also been shown in children with PAH although the measurements of RV end-diastolic dimension were measured from the parasternal short axis view from M-mode (6). Future studies are needed in RV basal diameters in children with PH to evaluate progressive RV dilation which is an early sign of RV dysfunction.

### Interventricular septum

**Figure 3:**
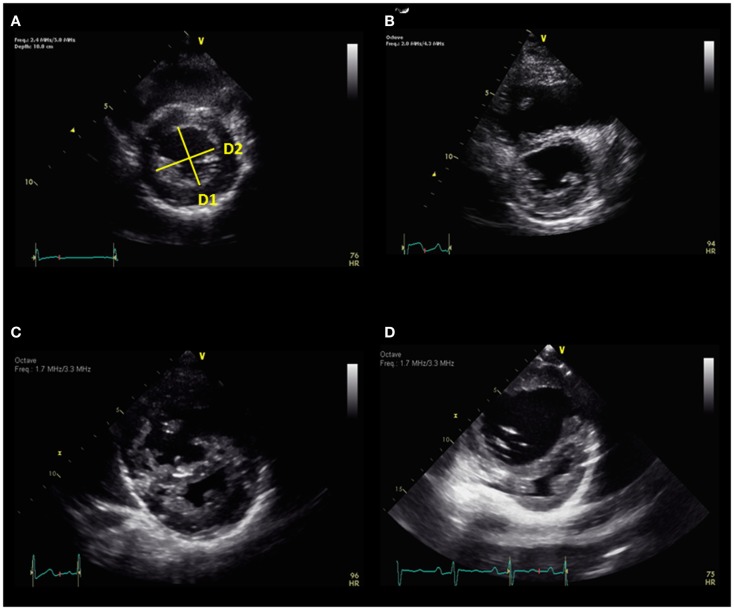
**(A)** Eccentricity index = D2/D1 and normal septum. There is progressive septal flattening from mild **(B)**, moderate **(C)**, to severe **(D)**. D1 = diameter 1, D2 = diameter 2.

**Figure 4:**
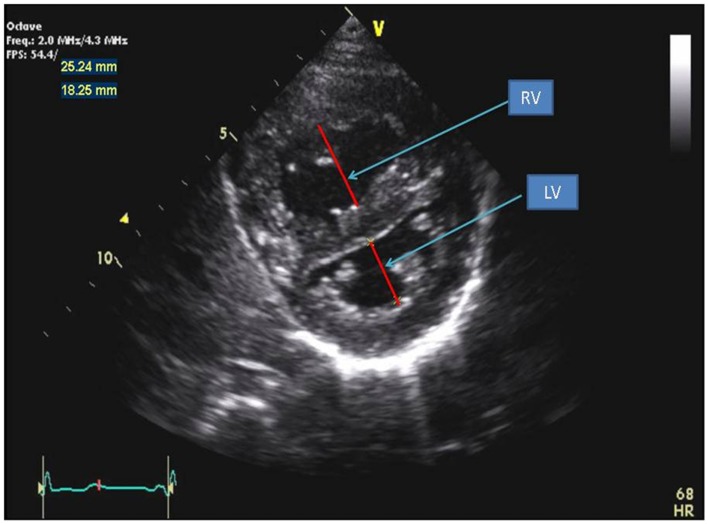
**Parasternal short axis view of the right and left ventricles at the level of the papillary muscles**. The RV/LV ratio is derived from RV diameter and LV diameter at end-systole.

Right ventricular pressure overload causes flattening of the interventricular septum (IVS) in end-systole into the left ventricle (LV), resulting a “D-shaped” LV in parasternal short axis view. Eccentricity index has been derived from the ratio between the LV anteroposterior dimension and the septolateral dimension at the level of the papillary muscle (Figure 3) (15). LV deformation of the IVS is greatest in end-systole in patients with RV pressure overload. Eccentricity index is abnormal when the ratio is >1.0 and has been shown to correlate well with invasive measurements of pulmonary artery pressure and associated with adverse clinical outcome in adults with PH (5, 16). Serial evaluation of eccentricity index with improvement in this index has been shown in targeted PH therapy in adults (17). The eccentricity index has been shown in children to be worse in patients with idiopathic PH compared to PH associated with congenital heart disease (6). Flattening of the IVS can be classified into mild, moderate, or severe depending on the degree of PH (Figure 3). In the absence of tricuspid regurgitation (TR) to estimate RV pressure, septal flattening offers indirect evidence of elevated pulmonary artery pressure. End-systolic flattening of the IVS has proven to be a sensitive marker for RV systolic hypertension in children (18). RV/LV ratio at end-systole measured at the level of the papillary muscles incorporates RV dimension in the parasternal short axis view and has been shown to correlate with invasive measures of hemodynamics and RV/LV end-systolic ratio >1 is associated with adverse clinical outcomes in children with PH (Figure 4) (19). Flattening of IVS into the LV impairs LV filling. Both the systolic and diastolic volumes are reduced. The importance of ventricular–ventricular interactions is increasingly recognized in patients with PH in both adult and pediatric populations (20, 21). Interventricular septal shift impairs LV diastolic filling, which results in decreased LV function. In severe PH with severe septal shift, the LV mid-cavity or outflow tract may become obstructed and the cardiac output (CO) can be decreased. CO can be estimated from echocardiography by the following equation:

$$\mathrm{CO}={\left(\mathrm{LVOT diameter}∕2\right)}^{2}\times 3.\mathrm{14}\times \mathrm{VTI}\;\left(\mathrm{LVOT}\right)\times \mathrm{HR}$$

The LV outflow tract (LVOT) is obtained from the parasternal long axis view and the velocity time integral (VTI) of LVOT is obtained by spectral Doppler from an apical four chamber view tilted anterior to view the LVOT. Cardiac index is calculated by dividing the CO by the body surface area.

### Atrial level shunt

An atrial level shunt is important in patients with PH as it provides relief to symptoms of severe PH by increasing systemic flow, reducing RV preload, and increasing CO. Atrial level shunts can be assessed in the parasternal short axis, subcostal short axis, and subcostal long axis views. Contrast echocardiography may also help in the assessment of atrial level shunt. In adults with PH, functional class has been shown to improve with the creation of an atrial level shunt, which leads to cyanosis from right to left shunting but can increase CO. Some series advocate use of an atrial septostomy to prolong survival (22, 23).

### Pericardial effusion

The presence of pericardial effusion is associated with increased risk of poor outcome in adults with PH but has not been a prognostic indicator for children (5). The size of the pericardial effusion is not predictive of outcome in children. Pericardial thickening and total pericardial score has been developed to score the amount of pericardial effusion seen in patients with PH (24). Increased pericardial thickening and increased pericardial effusion were found to be higher in adults with severe PH.

## Hemodynamic Assessment Using Doppler Echocardiography

### Systolic pulmonary artery pressure

The normal TR jet has a maximal velocity of <2.5 m/s (25). The normal estimated systolic pulmonary artery pressure (SPAP) is ≤35 mmHg (26). SPAP can be estimated from a peak TR velocity by continuous-wave Doppler using the modified Bernoulli equation in the absence of RV outflow tract (RVOT) obstruction (27). This is the most useful non-invasive method to predict SPAP. The mean RA pressure must be added to the result of the Bernoulli equation to determine the RV systolic pressure (RVSP). In the absence of RVOT obstruction, the SPAP equals the RVSP. The equation is below:

$$\mathrm{RVSP}=\mathrm{SPAP}=4{\left(\text{TR max}\right)}^{2}+\text{mean RA pressure }\left(\mathrm{mRAP}\right)$$

**Figure 5:**
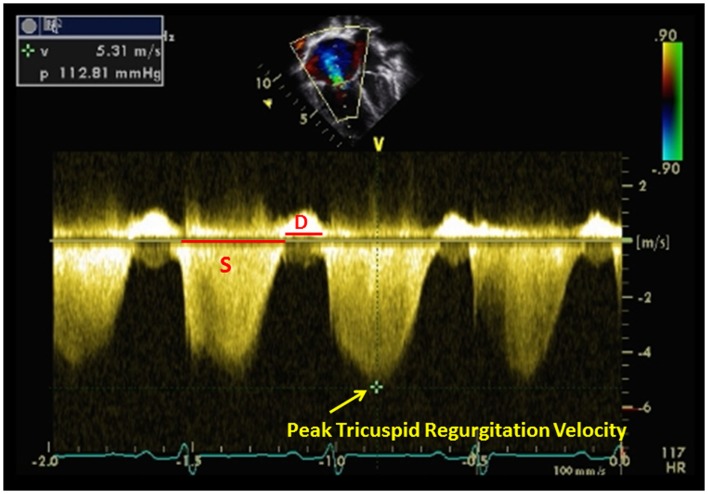
**Tricuspid regurgitation estimating right ventricular pressure in a patient with pulmonary hypertension**. Systolic (*S*) to diastolic (*D*) ratio from tricuspid regurgitation velocity can be measured.

Estimation of SPAP from the TR jet is dependent on the angle and the presence of the sufficient Doppler envelope (Figure 5). Therefore, it is recommended that the TR is obtained from multiple views (apical four chamber view or parasternal views) until the best Doppler angle and the highest velocity are obtained. Adult studies have shown that this method of estimating SPAP correlates linearly with hemodynamic assessment in cardiac catheterizations (28, 29). In pediatric patients with chronic lung disease, this estimate of SPAP using TR velocity has been shown to correctly diagnose the presence or absence of PH in 79% of children but was only able to diagnose correctly the severity of PH in 47%. (30) In a recent prospective trial of pediatric PH patients using TR to estimate SPAP compared to right heart catheterization, overestimation and underestimation of RV pressure occurred and TR velocity was inaccurate in children with elevated right heart pressures (31). Nevertheless, TR is still used clinically and serially to evaluate children with PH.

Estimation of SPAP can also be made in the presence of ventricular septal defect (VSD). This can only be done if there is no RV or LV outflow tract obstruction. This method has been shown to correlate well with invasive measurements obtained by cardiac catheterization (32, 33). The equation is below:

$$\begin{array}{l} \text{SPAP}=\text{Systolic blood pressure }(\text{SBP})-4V\;(\text{VSD})\;\max^{2}\\ \;(\text{for left to right shunts})\\ \text{SPAP}=\text{SBP}+4V\;(\text{VSD})\;\max^{2}\;(\text{for right to left shunts}) \end{array}$$

**Figure 6:**
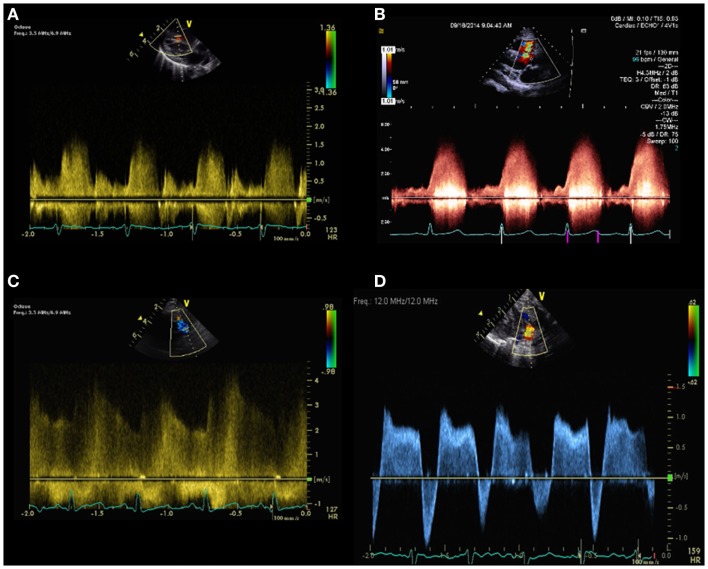
Spectral Doppler pattern across a ventricular septal defect with low velocity left to right shunt **(A)** indicating pulmonary hypertension and high velocity left to right shunt **(B)** indicating low pulmonary pressure. Spectral Doppler pattern across patent ductus arteriosus with continuous high velocity left to right shunt **(C)** indicating lower pulmonary artery pressure compared to aortic pressure and bidirectional shunt **(D)**, indicating pulmonary hypertension.

The maximal velocity across the ventricular septal defect is *V*(VSD)max. Flow direction and velocity across the VSD may help in the diagnosis of PH. Right to left shunt across the VSD and low velocity left to right shunt may suggest the presence of elevated pulmonary pressure (Figure 6). Parasternal and subcostal long axis views are used to interrogate perimembranous VSDs whereas parasternal and subcostal short axis views are best used to interrogate muscular VSDs.

Lastly, SPAP can be estimated in patients with patent ductus arteriosus (PDA) in the following equation:

$$\begin{array}{lll} \mathrm{SPAP} & =\mathrm{SBP}-4V\left(\mathrm{PDA}\right)\;\mathrm{ma}x^{2}\left(\text{for left to right shunts}\right)\; & \;\\ \mathrm{SPAP} & =\mathrm{SBP}+4V\left(\mathrm{PDA}\right)\;\mathrm{ma}x^{2}\left(\text{for right to left shunts}\right)\; & \; \end{array}$$

The maximal velocity across the PDA is denoted as *V*(PDA) max. This method has been validated by invasive measurements in cardiac catheterization (34). The flow and velocity across the PDA are dependent on the pressure between the aorta and the main pulmonary artery. Right to left shunting across the PDA indicates SPAP higher than the aortic pressure (Figure 6). Left to right shunting across the PDA indicates lower SPAP compared to the aortic pressure. Bidirectional shunting across the PDA is a common finding in newborns until the pulmonary vascular resistance (PVR) has decreased (Figure 6).

### Diastolic pulmonary artery pressure

**Figure 7:**
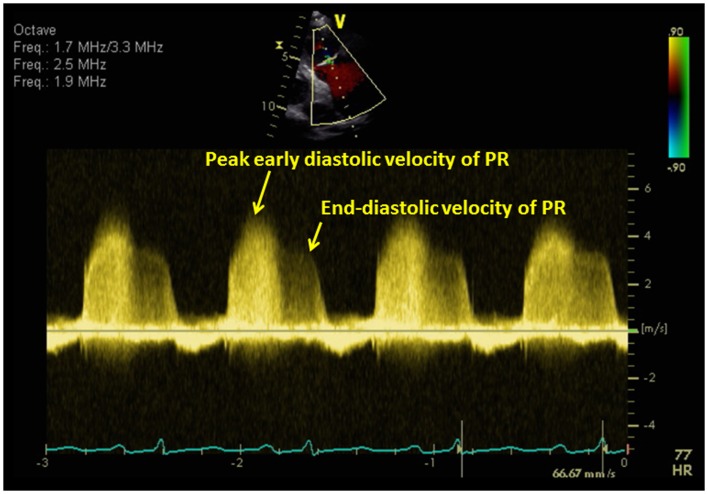
**The diastolic pulmonary artery pressure (DPAP) can be estimated from the velocity of the end-diastolic pulmonary regurgitant velocity using the modified Bernoulli equation**. The mean pulmonary artery pressure (mPAP) can be estimated from the following equation: mPAP = 4*V*(early peak pulmonary regurgitation velocity)^2^ + RA pressure.

The diastolic pulmonary artery pressure (DPAP) can be estimated from the velocity of the end-diastolic pulmonary regurgitant velocity using the modified Bernoulli equation (Figure 7):

$$\begin{array}{ll} \mathrm{DPAP}=4V{\left(\text{end-diastolic pulmonary regurgitation velocity}\right)}^{2}\\ \;+\text{ RA pressure}\; & \; \end{array}$$

### Mean pulmonary artery pressure

The mean pulmonary artery pressure (mPAP) can be estimated from the following equation (Figure 7):

$$\begin{array}{ll} \mathrm{mPAP}=4V{\left(\text{early peak pulmonary regurgitation velocity}\right)}^{2} & \\ \;+\text{ RA pressure} \end{array}$$

This equation has been shown to correlate well with invasive measurements in adults and children (35, 36). Another method to estimate mPAP is by using pulmonary acceleration time (AT) measured by pulsed-wave Doppler of the pulmonary artery in systole, where mPAP = 79 – (0.45 × AT) and in patients with AT < 120 ms, the formula for mPAP = 90 − (0.62 × AT) performed better (37). Recently, a newer method to evaluating mPAP is by adding the mean RA pressure to the velocity–time integral of the TR jet. This method has been validated by invasive measurements in adults where the mean difference between mPAP calculated using this method was closer to the right heart catheterization mPAP (38, 39). Some of the equations used to estimate mPAP do not add the mean RA pressure.

### Pulmonary vascular resistance

Pulmonary vascular resistance is calculated from cardiac catheterization as pressure gradient across the pulmonary bed divided by the pulmonary blood flow. PVR is important in the evaluation of PH and can be estimated using the following equation:

$$\mathrm{PVR}=\left[V\left(\mathrm{TR}\right)\;\max∕\mathrm{VTI}\left(\mathrm{RVOT}\right)\times 10\right]+0.\mathrm{16}$$

The VTI(RVOT) denotes the velocity time integral of RVOT that can be obtained by spectral Doppler in the parasternal short axis view. In adults, this echocardiographic derived PVR has been validated with cardiac catheterization (40). In another adult study that included children, PVR can be estimated using a simple ratio of peak TR velocity to the VTI(RVOT) and value of >38 provided a specificity of 100% for a PVR of >8 Wood units (WU) (41). However, this relationship is not reliable in patients with very high PVR as determined by invasive hemodynamic measurements (42). In children with congenital heart disease, the ratio of isovolumic time (IVRT) to RV ejection time (ET) has been shown to correlated with PVR, with IVRT/ET <0.3 being 97% specific for a PVR < 3 WU and a IVRT/ET ratio > 0.4 highly predictive of PVR > 5WU (43). However, other studies did not find a correlation between the ratio of IVRT/ET and PVR. Recently, Panda et al. used TR velocity over VTI(RVOT) ratio (TRV/VTIRVOT) to correlate with invasive measurements of PVR in children with PH in congenital heart disease (44). The TRV/VTIRVOT ratio correlated well with PVR measured at catheterization. They found that for PVR of 6 WU, a TRV/VTIRVOT value of 0.14 provided a sensitivity of 96.67% and a specificity of 92.86% and for PVR of 8 WU a TRV/VTIRVOT value of 0.17 provided a sensitivity of 79.17% and a specificity of 95% (44). Although echocardiogram can estimate PVR, cardiac catheterization remains the gold standard in diagnosing PVR.

## Other Doppler Echocardiography in Pulmonary Hypertension

With rising pulmonary artery pressure, the RV outflow spectral Doppler pattern changes from a smooth round shape to a triangular shape (45). RV AT is also decreased in patients with PH. The ratio of AT/ET < 0.3 is suggestive of PH but cannot reliably determine the severity of PH. Alkon et al. used a simple measure of systolic to diastolic time (*S*/*D*) ratio (Figure 5) from the TR jet to evaluate pediatric PH patients and found that as the RV function worsens, the systolic portion of the cardiac cycle lengthens leading to an increased *S*/*D* ratio (46). *S*/*D* ratio was found to be higher in pediatric PH patients compared to controls and is associated with worse hemodynamics by cardiac catheterization, shorter 6 min walk test, and worse clinical outcomes independent of PVR or pressures (46).

## Right Ventricular Systolic Function

The RV function has been shown to be an important prognostic determinant of PH. The assessment of RV function is more difficult because of its complex geometry and visual assessment of RV function is frequently used (47). The RV is composed of the smooth muscular inflow, the outflow region, and the trabecular apical region. The RV has inner longitudinal fibers that result in base to apex contraction and superficial circumferential muscle fibers responsible for its inward bellow movement (48). The evaluation of RV systolic function can be divided into global and regional systolic function.

### Global assessment of right ventricular function

#### RV fractional area change

The RV fractional area change (FAC) is a measure of RV systolic function and can be calculated by the following equation:

$$\begin{array}{ll} \mathrm{RVFAC}=\left(\text{end-diastolic area-end-systolic area}\right)∕\; & \;\\ \;\text{end-diastolic area}\times \mathrm{100}\; & \; \end{array}$$

**Figure 8:**
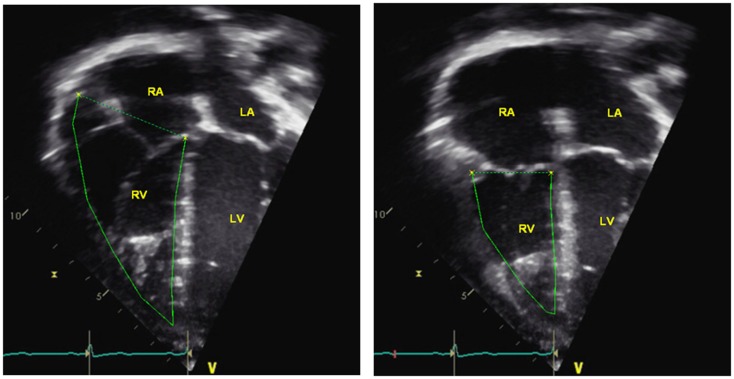
**Right ventricular fractional area change = (end-diastolic area – end-systolic area)/end-diastolic area × 100**. RA = right atria, RV = right ventricle, LA = left atria, LV = left ventricle.

The RV FAC can be obtained by tracing the RV endocardium both in systole and diastole from the tricuspid valve annulus, along the right free wall to the apex, and then back to the tricuspid valve annulus, along the IVS (Figure 8). RV FAC has been shown to correlate with RV ejection fraction (EF) by magnetic resonance imaging (MRI) (49). In adults, the lower reference value for normal RV FAC is 35%. In pediatric patients with idiopathic PAH, RV FAC was found to be significantly worse in patient who did not survive at follow-up when compared to patients who survived (6).

#### Three-dimensional volume and EF estimation

**Figure 9:**
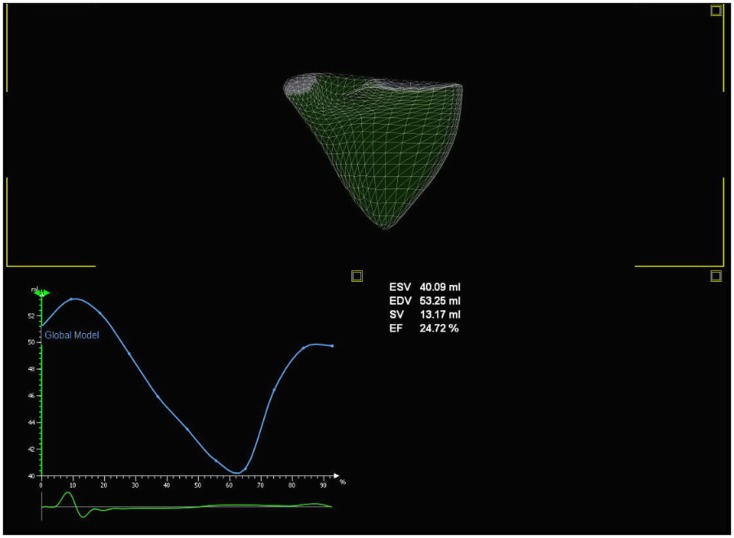
**Three-dimensional echocardiography evaluation of the right heart volume and ejection fraction**.

By eliminating the need for geometric assumptions, three-dimensional (3D) echocardiography provides more accurate and reproducible measurements of RV volume and EF in adults with PH (Figure 9) (50). Disk summation and apical rotational methods for RV volume and EF calculation are the most commonly used methods in 3D echocardiography. Both methods have been shown to correlate well with MRI volume and EF in children (51–54) and in adults (55–61). The lower reference limit for RV EF is 44% from disk summation method in adult patients. In a recent study by Kong et al. regional and global RV systolic dysfunction in adult PH patients measured by 3D echocardiography were inversely related to the PASP and PVR (62). Measurements of volumes and EF become difficult in patients with arrhythmia and breath holding in small children maybe a limiting factor; however, with the advent of newer software using semi-automated quantification of RV volume and EF, breath holding is no longer required. RV volume and EF from 3D echocardiography can be accurately measured using real-time 3D algorithms and need further studies in children with PH.

### Myocardial performance index

The myocardial performance index (MPI) is a non-invasive measurement of global ventricular function (systolic and diastolic) independent of geometric assumptions (63). It can be applied to the RV and the LV. It is defined as the ratio of isovolumic time divided by the ET.

$$\mathrm{MPI}=\left(\text{isovolumic contraction time}\left(\mathrm{IVCT}\right)+\mathrm{IVRT}\right)∕\mathrm{ET}$$

**Figure 10:**
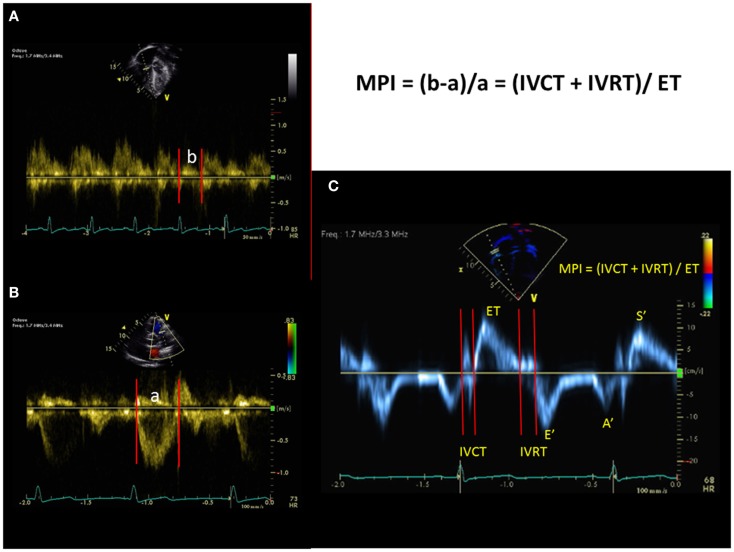
**The myocardial performance index (MPI) can be obtained from the spectral Doppler of tricuspid inflow and pulmonary outflow velocities [top left and bottom left, (A,B)] or can be obtained from tissue Doppler imaging (C) at the lateral annulus of the tricuspid valve**. IVCT = isovolumic contraction time, IVRT = isovolumic relation time, and ET = ejection time. Tissue Doppler imaging of the tricuspid valve. *S′*= systolic velocity, *E′* = early diastolic velocity, *A′* = late diastolic velocity.

The MPI is easily obtained from either the spectral Doppler or from tissue Doppler imaging (TDI) (Figure 10). RV MPI is difficult to obtain in one single heart beat or similar heart beat because it is difficult to image the tricuspid valve and pulmonary valve in the same spectral Doppler signal. This results in errors in the calculation of the RV MPI. In TDI, the RV MPI is more easily measured from a single heart beat by sampling at the tricuspid annulus. RV MPI using the spectral Doppler has been studied in pediatric patients with PH. It is increased in PH patients compared to controls and RV MPI correlated well with both mPAP at cardiac catheterization and response to therapy. (64) Another study in infants with PH from congenital diaphragmatic hernia has shown that RV MPI is elevated compared to controls, but did not correlate well with SPAP (65). RV MPI may be a useful tool to follow PH patients serially but it may be unreliable if there is significant TR with an elevated RA pressure because the IVRT will shorten and result in an inappropriately lower value of MPI. RV MPI will not work if patients have irregular heart rates. There are currently no studies in children using TDI method of RV MPI to evaluate pediatric PH and further studies are needed in this area.

### Regional assessment of right ventricular function

#### Tricuspid annular plane systolic excursion

**Figure 11:**
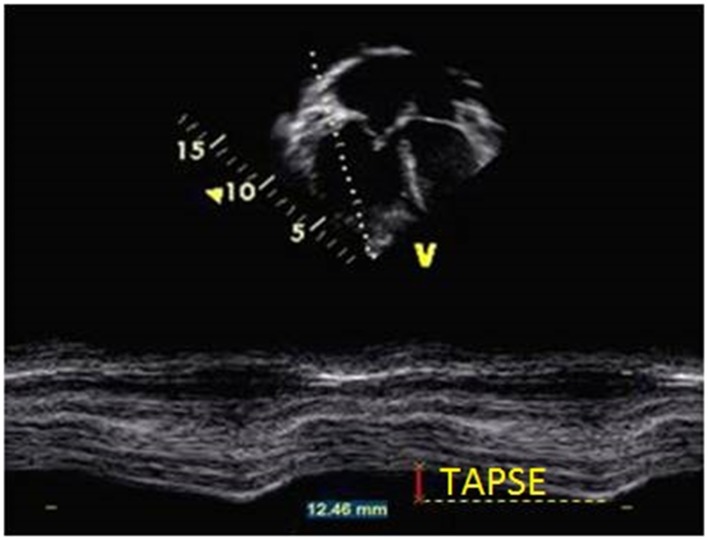
**Measurement of tricuspid annular plane systolic excursion (TAPSE) from M-mode in the apical four-chamber view at the level of the tricuspid annulus in a patient with pulmonary hypertension**.

The longitudinal fibers are the major contributor to RV systolic function. Tricuspid annular plane systolic excursion (TAPSE) is a method to measure the distance of systolic excursion of the RV annular segment along its longitudinal plane from the apical four chamber view in millimeters from end-diastole to end-systole. The greater the descent of the basal annulus in systole, the better RV systolic function is. TAPSE is usually acquired by placing the M-mode cursor through the lateral tricuspid annulus and measuring the amount of longitudinal motion of the annulus in peak systole (Figure 11). TAPSE < 18 mm has been demonstrated in adult patients with PH to be associated with greater RV systolic dysfunction and lower survival rate (66). There is also a strong correlation between TAPSE and reduced RV FAC regardless of pulmonary artery pressures in adults (67). Normal values for TAPSE in children have been published and *z*-scores established (68). Children with idiopathic PAH who had TAPSE *z*-score <−4.3 were associated with increased risk of transplant or death (6). The advantage of using TAPSE is that it is a simple and reproducible measure but it is important to note that TAPSE is angle and load dependent. TAPSE is also one-dimensional and does not take into the account the three-dimensional structure of the RV. TAPSE does not evaluate apical systolic dysfunction in patients with PH.

#### Tissue Doppler imaging

Tissue Doppler imaging measures the myocardial velocities in the apical four chamber view with the pulse-wave Doppler sample volume placed at the level of RV lateral tricuspid, basal IVS, and LV lateral mitral annuli (Figure 10). Myocardial systolic wave (*S*′) measures the systolic longitudinal function of the RV or the LV and two diastolic waves: early diastolic (*E*′) and late diastolic (*A*′) denote the diastolic function of the ventricles. Pulse-wave TDI measures the peak myocardial velocities and color TDI measures the mean velocities, which is lower than the pulse-wave TDI measurements by 20%. Acquisition of the TDI velocities must be as parallel as possible to avoid underestimation of the velocities. In adults with idiopathic PH, tricuspid and septal peak systolic (*S*′) velocities relate to the RV dysfunction and cut-off values have been described to predict RV systolic dysfunction (69). RV *S*′ at the tricuspid valve has also been shown to have an inverse relationship with cardiac catheterization mPAP and PVR in adults. (70) RV *S*′ at the tricuspid valve has been shown to correlate with RV FAC and TAPSE (67). The lower limit value of RV *S*′ at the tricuspid valve in adults is <10 cm/s (3). A strong correlation has been shown between RV *S*′ at the tricuspid valve and RV EF with a RV *S*′ < 10 cm/s predicting RV EF < 40% (71, 72). In infants with PH, RV *S*′ at the tricuspid valve is lower compared to controls (73). RV *S*′ by pulsed Doppler is a simple, reproducible measure of basal RV free wall function and should be used in the assessment of RV regional systolic function.

#### Strain and strain rate

**Figure 12:**
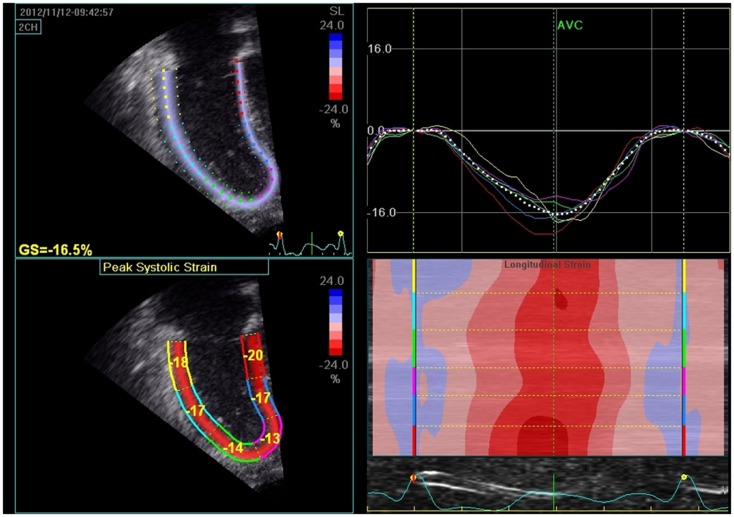
**The left upper panel shows 2D strain (speckle tracking) of right ventricle in pulmonary hypertension patient. The right upper panel represents the strain curves sampled in each of the analyzed myocardial segments and decreased global longitudinal strain (GS = −16.5%). The lower left panel measures the peak systolic strain in each of the myocardial segments. The lower right panel depicts the longitudinal strain according to the color map of M-mode; red segments indicate higher contractility**.

Strain measures the percentage change in myocardial deformation whereas its time derivative, strain rate, defines the rate of deformation of myocardium over time. Strain is load-independent global measure of ventricular systolic function and correlates closely with myocardial contractility (Figure 12). Strain can measure regional and global systolic function. Using speckle tracking, longitudinal strain is displayed as a negative wave. For the RV, longitudinal and radial strain and strain rate are typically assessed. Optimal quality images with frame rates between 60 and 90 frames/s during image acquisition are necessary prerequisites for reproducibility and reliability of the data. RV free wall strain imaging has been applied to adult patients with PH and has been shown to correlate with invasive pulmonary hemodynamics (74). Recently, Fine et al. demonstrated that RV longitudinal strain is a powerful tool to predict clinical outcome in adults with PH (75). Smith et al. demonstrated that 3D area strain, radial strain, longitudinal strain, and circumferential strain were lower in adult PH patients compared to controls and reduced area strain, longitudinal strain, circumferential strain, and EF were all determinants of mortality in adults with PH (76). The disadvantages of strain are that there are many different vendors with strain software and no standard methods of measuring strain. Some are deriving strain and strain rate from velocity vector imaging (VVI) and others derive it from speckle tracking. Normal values for the RV and LV strain have been published in children (77). Normal ranges of RV systolic and diastolic strain measures in children using speckle tracking have been published in a meta-analysis (78). Evaluation of 2D RV strain in children with PH is promising but requires further research. 3D strain has not been investigated in children and will require further research.

## Right Ventricular Diastolic Function

Pulmonary hypertension can result in RV diastolic dysfunction, which affects outcome in these patients. In the apical four chamber view, a pulsed Doppler beam should be aligned as parallel as possible to RV inflow. The sample volume should be placed at the tip of the tricuspid leaflets (79). Tricuspid inflow can be achieved with high reproducibility (80). The presence of moderate to severe TR can confound measurements of the tricuspid inflow velocities (*E* and *A*) and are excluded from most studies. The evaluation of RV diastolic function includes tricuspid inflow velocities (*E*, *A*, *E*/*A*), TDI of the tricuspid annulus (*E*′, *A*′, and *E*′/*A*′), deceleration time, and IVRT. The tricuspid *E*/*E*′ ratio, RA area, and diastolic strain rate have shown promise in the evaluation of RV diastolic function. In adult studies with chronic heart failure and PH, the presence of RV diastolic dysfunction is associated with worse functional class and is an independent predictor of mortality (81). In infants with PH, diastolic *E*′ velocities have been shown to be lower than controls (73). In pediatric patients with bronchopulmonary dysplasia, increasing tricuspid *E*/*E*′ has been shown to correlate with clinical severity of the disease (82). In children with idiopathic PAH, tricuspid valve *E*′ correlated with mPAP and RV end-diastolic pressure (83). In this study, the tricuspid valve *E*′ was lower in worse functional class and the cumulative event-free survival rate was significantly lower when tricuspid valve *E*′ was <8 cm/s (83). In a recent study, echocardiographic diastolic parameters of RV in children with PAH correlated with invasive measures of cardiac catheterization and tricuspid deceleration time with global early diastolic strain rate were independent predictors of tau (84).

## Left Ventricular Diastolic and Systolic Function

In PH patients, the late diastolic filling pattern through the mitral valve will reverse with *E* < *A*, *E*/*A* < 1, and a short *E* deceleration time have been observed in adults (85). The pulmonary venous Doppler can be abnormal in patients with LV diastolic dysfunction. In patient with PH from left sided heart disease, the evaluation of mitral valve and pulmonary veins remains important in determining the cause of PH. Evaluation of the LV systolic function can be measured by EF from bi-plane Simpson’s formula (86, 87).

## Conclusion

Echocardiography is a valuable non-invasive tool in screening, diagnosing, and assessing pediatric PH. Echocardiography provides indirect measurements of pulmonary artery pressures, which can help in the initial assessment and follow-up of these patients. Echocardiographic assessment of RV function is important in this population and can be used as a chronic surveillance of these patients. Advancement in technology has allowed new techniques in evaluating RV function (3D echocardiography, strain, and strain rate) using echocardiography but more data are needed in the pediatric population in the reproducibility of these new techniques.

## Conflict of Interest Statement

The authors declare that the research was conducted in the absence of any commercial or financial relationships that could be construed as a potential conflict of interest. The Review Editor Jeffrey Feinstein declares that despite having collaborated with author Dr D. Dunbar Ivy, the review process was handled objectively and no conflict of interest exists.
